# Tirzepatide Attenuates Wire Injury-Induced Arterial Remodeling in Non-Diabetic and Diabetic Mice: Comparison with Semaglutide

**DOI:** 10.3390/biomedicines14071554

**Published:** 2026-07-11

**Authors:** Yusaku Mori, Naoya Osaka, Michishige Terasaki, Hironori Yashima, Tomomi Saito, Daiki Tanno, Madoka Ogino, Makoto Ohara, Sho-Ichi Yamagishi

**Affiliations:** Department of Diabetes, Metabolism, and Endocrinology, Showa Medical University Graduate School of Medicine, 1-5-8 Hatanodai, Shinagawa, Tokyo 142-8555, Japanttmichi@med.showa-u.ac.jp (M.T.);

**Keywords:** arterial remodeling, diabetes, dual GIP/GLP-1 receptor agonist, GLP-1 receptor agonist, nitric oxide, vascular endothelial cells

## Abstract

**Background**: Glucose-dependent insulinotropic polypeptide receptor (GIPR) and glucagon-like peptide-1 receptor (GLP-1R) activation exert anti-diabetic and anti-obesity effects. Tirzepatide, a dual GIPR/GLP-1R agonist, has demonstrated cardiovascular benefits in clinical studies. However, the direct vascular actions of tirzepatide and their potential advantages over selective GLP-1 receptor agonists (GLP-1RAs) remain unclear. We investigated the vasoprotective effects of tirzepatide and compared them with those of GLP-1 receptor agonists in vivo and in vitro. **Methods**: Non-diabetic C57BL/6 and diabetic KK-Ay mice received tirzepatide, semaglutide, or vehicle. Arterial remodeling was induced by femoral artery wire injury. A subset of mice was co-treated with the nitric oxide synthase inhibitor Nω-nitro-L-arginine methyl ester (L-NAME). After 4 weeks, biochemical, morphometric, and immunofluorescence analyses were performed. In vitro, human umbilical vein endothelial cells (HUVECs) were stimulated with tirzepatide or liraglutide to assess nitric oxide (NO) production. **Results**: In non-diabetic mice, tirzepatide suppressed intimal hyperplasia, including at a low dose that did not affect metabolic parameters, whereas semaglutide had no significant effect on intimal hyperplasia at the same molar dose. The protective effects of tirzepatide were abolished by L-NAME. In diabetic mice, tirzepatide and semaglutide similarly improved metabolic parameters and attenuated intimal hyperplasia. In HUVECs, tirzepatide increased NO production in a dose-dependent manner, and this effect was preserved under hyperglycemic conditions. Tirzepatide and liraglutide induced comparable NO production at equivalent molar concentrations. **Conclusions**: Tirzepatide, but not semaglutide, exerted vasoprotective effects under non-diabetic conditions in a NO-dependent manner, whereas both agents exhibited comparable vasoprotective effects under diabetic conditions.

## 1. Introduction

The prevalence of type 2 diabetes has been rising worldwide [1]. Type 2 diabetes is a well-established risk factor for atherosclerotic cardiovascular disease (ASCVD), which remains a leading cause of mortality in individuals with type 2 diabetes [2]. Preventing ASCVD is therefore a key clinical priority in the management of type 2 diabetes. However, large-scale clinical trials have demonstrated that the benefits of intensive glycemic control on ASCVD prevention are limited [3]. These observations highlight the need to develop therapeutic strategies beyond glucose lowering in individuals with type 2 diabetes.

Glucagon-like peptide-1 (GLP-1) and glucose-dependent insulinotropic polypeptide (GIP), collectively known as incretins, are peptide hormones that are secreted from intestinal cells in response to nutrient intake [4]. Both incretins stimulate insulin secretion from pancreatic β-cells in a glucose concentration-dependent manner, thereby minimizing the risk of hypoglycemia. Furthermore, GLP-1 receptor (GLP-1R) and GIP receptor (GIPR) are expressed in extra-pancreatic tissues, including the central nervous system, where their activation suppresses appetite and subsequently promotes body weight reduction [4].

Importantly, our previous studies demonstrated that activation of GLP-1R and GIPR attenuates arterial remodeling and atherosclerosis independently of glucose lowering in experimental models of cardiovascular disease [5,6,7,8,9]. Consistent with these findings, large-scale clinical trials have shown that treatment with GLP-1 receptor agonists (GLP-1RAs) reduces cardiovascular events in individuals with type 2 diabetes at high ASCVD risk [10]. Accordingly, GLP-1RAs are now regarded as important therapeutic agents in the management of type 2 diabetes, owing to their glucose- and body weight-lowering effects, low risk of hypoglycemia, and additional cardiovascular benefits [11,12,13].

More recently, tirzepatide, a dual GIPR/GLP-1R agonist, has emerged as a next-generation incretin-based therapy [14]. Clinical trials have demonstrated the superior glucose- and body weight-lowering efficacy of tirzepatide compared with GLP-1 receptor agonists [15,16]. Furthermore, recent cardiovascular outcome data support the clinical cardiovascular benefit of tirzepatide in individuals with type 2 diabetes and established ASCVD [17]. However, whether tirzepatide exerts direct vasoprotective effects beyond its metabolic actions and whether these effects differ from those of selective GLP-1R agonists remain unclear.

In the present study, we therefore investigated whether tirzepatide suppresses arterial remodeling and whether its vasoprotective effects differ from those of semaglutide at equivalent molar doses. We further explored the contribution of endothelial nitric oxide signaling using pharmacological inhibition in vivo and cultured vascular endothelial cells (VECs) in vitro. To our knowledge, this is the first study to demonstrate that tirzepatide attenuates arterial remodeling after vascular injury through an NO-dependent mechanism, at least partly independent of glucose reduction.

## 2. Materials and Methods

### 2.1. Chemical Agents

The following chemical agents were used: tirzepatide (Eli Lilly and Company, Indianapolis, IN, USA); semaglutide (Novo Nordisk, Bagsværd, Denmark); Nω-nitro-L-arginine methyl ester (L-NAME) and basic fibroblast growth factor (bFGF) (Fujifilm Wako Pure Chemical Corporation, Minato, Tokyo, Japan); tirzepatide sodium salt, SQ22536, U73122, Akt inhibitor X, and dorsomorphin dihydrochloride (Cayman Chemical, Ann Arbor, MI, USA); PKI (14–22) amide, myristoylated (Sigma-Aldrich Japan, Minato, Tokyo, Japan); and liraglutide (Adipogen Life Sciences, San Diego, CA, USA).

### 2.2. Animal Experiments

The study protocol was approved by the Animal Care Committee of Showa Medical University School of Medicine (approval number and date: 124004 and 1 April 2024). Animal experiments were conducted in accordance with ARRIVE 2.0 guidelines and the Guide for the Care and Use of Laboratory Animals (8th Edition) [18,19]. All invasive procedures were performed under general anesthesia using isoflurane, except for plasma glucose measurements, in which blood samples were obtained via a small tail prick. Ten-week-old male C57BL/6 mice (non-diabetic, *n* = 80) and KK-Ay mice (diabetic, *n* = 18) were purchased from Nihon Clea (Meguro, Tokyo, Japan). Based on an expected mean difference of 1.0 and a standard deviation of 0.4 [6,7,20], a three-group comparison with equal allocation, α = 0.05, and 80% power using Tukey’s multiple-comparison test indicated that approximately five animals per group would be required. In Experiment 1, four groups were compared in a dose-finding setting. The treatment groups included 7–8 animals each, which exceeded the estimated requirement, and the vehicle group included 14 animals, allowing a more stable estimate of the control response. The sample size was also determined with consideration of the expected variability of the mouse femoral artery wire injury model and the ethical requirement to minimize animal use. Animals were housed in individual cages and provided standard chow (Labo MR Stock, NOSAN, Yokohama, Japan) with ad libitum access to food and water. All animals were maintained in a specific pathogen-free facility at the Division of Animal Experimentation, Showa Medical University School of Medicine. The rooms were maintained on a 12 h dark/light cycle, 21 °C, and 40–60% humidity. Following a 2-week acclimatization period, mice were allocated to each experimental group by sequential assignment according to cage number as follows (Day 1):

- Experiment 1: non-diabetic mice treated with vehicle or tirzepatide (0.16, 1.0, or 6.0 nmol/kg/day);
- Experiment 2: non-diabetic mice treated with L-NAME alone or L-NAME + tirzepatide (1.0 nmol/kg/day);
- Experiment 3: non-diabetic mice treated with vehicle, semaglutide (1.0 nmol/kg/day), or tirzepatide (1.0 nmol/kg/day);
- Experiment 4: diabetic mice treated with vehicle, semaglutide (1.0 nmol/kg/day), or tirzepatide (1.0 nmol/kg/day).

Tirzepatide and semaglutide were continuously administered from Day 1 using subcutaneously implanted osmotic pumps (Alzet minipump 1004; Cupertino, CA, USA). Tirzepatide doses were determined based on previous studies [21,22,23], and semaglutide was given at a molar dose equivalent to tirzepatide [24]. L-NAME was added to drinking water (100 mg/L), which has been previously shown to reduce plasma nitric oxide (NO) and aortic endothelial NO synthase (eNOS) phosphorylation in mice [6,7]. Femoral artery wire injury was performed on Days 3–5, as previously described [6,7]. In brief, a straight spring wire was retrogradely inserted into the femoral artery via a small incision on the muscle branch and withdrawn after 1 min. Mice were euthanized by inhaled isoflurane overdose, and injured arteries were harvested on Days 24–26 for morphometric and immunofluorescence analyses. All analyses were performed by investigators blinded to treatment allocation (N.O., H.Y., T.S., D.T., and M. Ogino). Animals with complete thrombotic occlusion of the injured femoral artery were excluded from the analysis according to predefined exclusion criteria. Animals were monitored regularly throughout the study. No unexpected adverse events were observed, and no predefined humane endpoints were reached during the study.

### 2.3. Measurement of Biochemical Parameters, Blood Pressure, and Pulse Rates

At the end of each experiment, blood samples were collected after 6–8 h of fasting. Plasma glucose, lipid, and insulin levels were measured using an enzymatic electrode method (StatStrip 2; Nipro, Settsu, Osaka, Japan), an enzymatic colorimetric assay (Fujifilm Wako Pure Chemical Corporation), and an enzyme-linked immunosorbent assay (Ultrasensitive Plus mouse insulin measurement kit; ID: M1105; Morinaga, Yokohama, Kanagawa, Japan), respectively. Homeostasis model assessment of β-cell function (HOMA-β) and insulin resistance (HOMA-IR) were calculated using the equations of Matthews et al. [25]. Blood pressure and pulse rates were measured on Days 23–25 using a non-invasive tail-cuff method (Model MK-2000ST; Muromachi Kikai, Chuo, Tokyo, Japan) [6,7]. The mean of 3–5 consecutive measurements was used as a single data point.

### 2.4. Morphometric Analysis

Cross-sections were taken every 0.5 mm from the proximal end of the femoral arteries, and the mean of 5 sections was used per artery to reduce selection bias [6,7]. Sections were stained with Elastica van Gieson (EVG), imaged with an inverted microscope (Model IX71, Olympus, Shinjuku, Tokyo, Japan), and analyzed using ImageJ software version 1.54h.(National Institutes of Health, Bethesda, MD, USA) by an investigator who was blinded to the treatment. The intima, media, and arterial perimeter were defined as the areas between the lumen–internal elastic lamina (IEL), the IEL–external elastic lamina (EEL), and the EEL circumference, respectively. Arteries with complete thrombotic occlusion, sections exhibiting disrupted wall structure, branching from another artery, or absence of clearly identifiable EL were excluded from the analysis, as previously described [6,7,20]. Approximately 5–15% of arteries and 20–30% of sections were excluded.

### 2.5. Immunofluorescence Staining

Immunofluorescence staining was performed as previously described [20]. Cross-sections were incubated overnight with anti-Ki67 antibody (Product ID: MA5-14520, RRID: AB_10979488, raised in rabbit, 1:250; Invitrogen, Waltham, MA, USA). Sections were then incubated with an appropriate secondary antibody for 4 h, counterstained with 4′,6-diamidino-2-phenylindole (DAPI), and mounted with VECTASHIELD Antifade Mounting Medium (Vector Laboratories, Newark, CA, USA). Immunofluorescence images were captured with a confocal microscope (BZ-X710; Keyence, Osaka-shi, Osaka, Japan) and analyzed with ImageJ software.

### 2.6. Cell Culture

Human umbilical vein endothelial cells (HUVECs; umbilical vein from pooled donors; authenticated by expression of CD31, CD105, Axl, eNOS, Tie-2, and vascular endothelial growth factor receptor 2) were obtained from Lonza Japan (C2519AS; Chuo, Tokyo, Japan) and cultured in EGM-2 medium (Lonza Japan). The following inhibitors were used at the indicated concentrations: adenylate cyclase (AC) inhibitor SQ22536 (100 μmol/L); protein kinase A (PKA) inhibitor PKI (14–22) amide, myristoylated (10 μmol/L); phospholipase C (PLC) inhibitor U73122 (7.17 μmol/L); AMP-activated protein kinase (AMPK) inhibitor dorsomorphin (1 μmol/L); and Akt inhibitor X (1 nmol/L) [6,7,26,27]. Because semaglutide showed limited aqueous solubility under our experimental conditions, liraglutide, a well-established GLP-1 receptor agonist with sufficient solubility, was used as a representative GLP-1 receptor agonist for in vitro experiments. For high-glucose experiments, cells were exposed to 20 mmol/L glucose for 24 h before stimulation. Cells between passages 4–9 were used in all experiments.

### 2.7. Measurement of NO Production

NO production was measured using DAX-J2 Red (AAA Bioquest, Pleasanton, CA, USA) according to the manufacturer’s instructions. HUVECs were seeded onto 24-well collagen-coated plates and grown to approximately 80% confluence. Growth medium was then replaced with M199 containing 2% fetal bovine serum (FBS) and 5 ng/mL bFGF. Following 1 h preincubation with vehicle or inhibitors, cells were stimulated with vehicle, tirzepatide sodium salt, or liraglutide at the indicated concentrations for 3 h. Cells were further incubated with DAX-J2 Red (5 μmol/L) for 30 min, washed twice with PBS, and imaged at 40× magnification using a BZ-X710 confocal microscope (Keyence). For each well, fluorescence intensity was quantified from three randomly selected fields and averaged.

### 2.8. Western Blotting

Western blotting was performed as described previously [6,7], with some modifications. After serum starvation for 6 h in 0.5% FBS-containing M199, HUVECs were stimulated with 3 μM tirzepatide for different time periods. The immunoblot membrane was incubated with the primary antibodies for 16 h and with the secondary antibodies for 1 h. The following antibodies were used: phosphorylated AMPKα at Thr 172 (p-AMPK; 1:2500; molecular weight 62; RRID: AB_331250; catalog no. 2535; Cell Signaling Technology Japan, Chiyoda, Tokyo, Japan); total AMPKα (1:5000; molecular weight 62; RRID: AB_330331; catalog no. 2532; Cell Signaling Technology Japan); p-endothelial NOS at Ser 1177 (p-eNOS; 1:3000; molecular weight 140; catalog no. 9570; RRID: AB_823493; Cell Signaling Technology Japan); total eNOS (1:5000; molecular weight 140; catalog no. 32027; RRID: AB_2728756; Cell Signaling Technology Japan); p-Akt at Ser473 (p-Akt; 1:2000; molecular weight 60; catalog no. 4060; RRID: AB_2315049; Cell Signaling Technology Japan); and β-actin (1:8000; molecular weight 42; catalog no. A2066; RRID: AB_476693; Sigma-Aldrich Japan). The bands on the immunoblot were detected using the Amersham ECL Prime kit (GE HealthCare Japan, Hino, Tokyo, Japan), digitized using a WSE-6100 LuminoGraph (ATTO, Taito, Tokyo, Japan), and quantified using CS Analyzer software (version 4; ATTO).

### 2.9. Statistical Analysis

Data are presented as means ± standard deviation (SD). Given the small sample sizes, formal tests for data normality and homogeneity of variance were not used as decision rules before applying parametric analyses, because these tests have limited power in small experimental groups. Two-group comparisons were performed using unpaired *t*-tests, and ≥3 group comparisons were performed with one-way ANOVA, followed by Tukey’s test. Correlations and dose–response relationships were assessed using Pearson’s correlation and the Jonckheere–Terpstra trend test, respectively. Statistical analyses were performed using JMP Pro software (version 17; SAS Institute, Cary, NC, USA), except for the Jonckheere–Terpstra trend test, which was conducted in EZR software (version 1.68) [28]. A *p*-value < 0.05 was considered statistically significant.

## 3. Results

### 3.1. Tirzepatide Suppressed Intimal Hyperplasia and Cell Proliferation After Wire Injury in Non-Diabetic Mice

**Figure 1:**
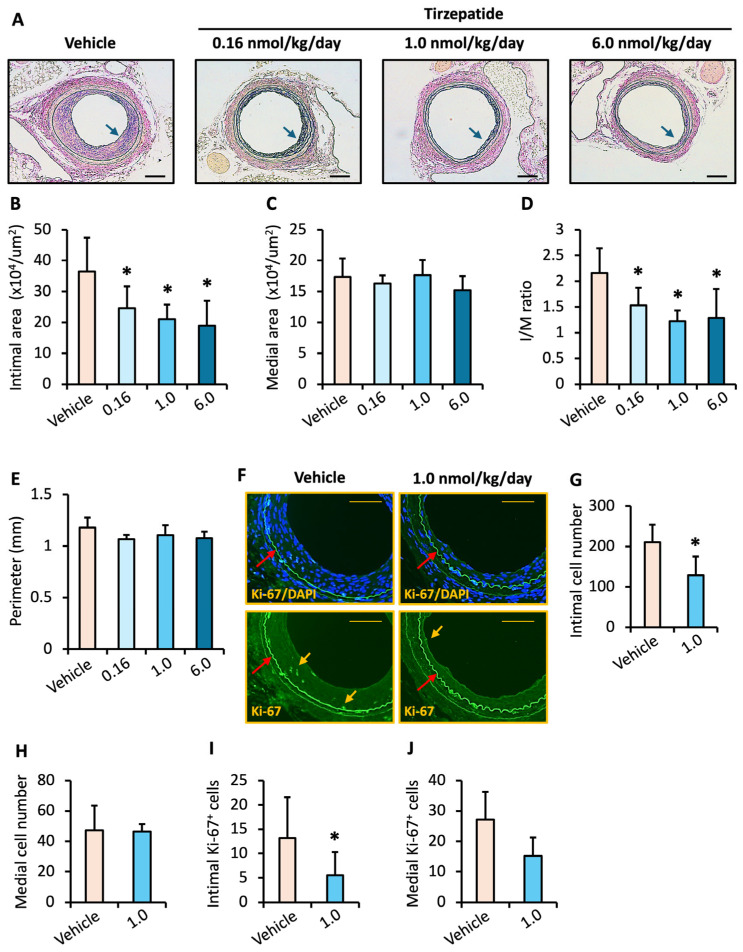
**Effects of tirzepatide at different doses on arterial remodeling in non-diabetic mice.** Injured arteries were harvested from non-diabetic mice treated with vehicle or tirzepatide at the indicated doses, 24–26 days after wire injury: (**A**) Representative images of EVG-stained femoral arteries. Arrows indicate the intima. Scale bar = 100 μm. (**B**) Intimal area. (**C**) Medial area. (**D**) Intima/media (I/M) ratio. (**E**) Arterial perimeter. (**B**–**E**) Vehicle, *n* = 14; tirzepatide 0.16 nmol/kg/day, *n* = 8; tirzepatide 1.0 nmol/kg/day, *n* = 7; tirzepatide 6.0 nmol/kg/day, *n* = 7. (**F**) Representative images of femoral arteries immunostained for Ki-67 (green). Nuclei were counterstained with DAPI (blue). Yellow arrows indicate Ki-67-positive cells, and red arrows indicate the internal elastic lamina. Scale bar = 100 μm. (**G**,**H**) Total cell number in the intima and media, respectively. (**I**,**J**) Number of Ki-67-positive cells in the intima and media, respectively. (**G**–**J**) Vehicle, *n* = 4; tirzepatide 1 nmol/kg/day, *n* = 4. Data are expressed as means ± SD. * *p* < 0.05 vs. vehicle group. Tirzepatide doses are shown as nmol/kg/day.

First, the effects of tirzepatide on arterial remodeling induced by wire injury were examined in non-diabetic mice. Physiological and biochemical parameters are summarized in Table 1. Tirzepatide at 1.0 and 6.0 nmol/kg/day significantly reduced fasting plasma glucose levels compared with the vehicle, whereas the 0.16 nmol/kg/day dose had no effect. Arterial morphological changes are presented in Figure 1. All doses of tirzepatide significantly decreased intimal area (Figure 1B) without affecting medial area or arterial perimeter, resulting in a reduced intima/media (I/M) ratio (Figure 1C–E). At 1.0 nmol/kg/day, tirzepatide also significantly reduced the numbers of total and proliferating cells in the intima but not in the media (Figure 1G–J), whereas cell densities in both layers remained unchanged (Supplemental Figure S1A,B). Among vascular parameters, the numbers of total and proliferating cells in the intima were significantly correlated with intimal area (Supplemental Table S1). The mean differences with 95% confidence intervals for the key vascular outcomes are summarized in Supplemental Table S2.

### 3.2. Co-Treatment with L-NAME Abolished the Protective Effects of Tirzepatide Against Arterial Remodeling in Non-Diabetic Mice

**Figure 2:**
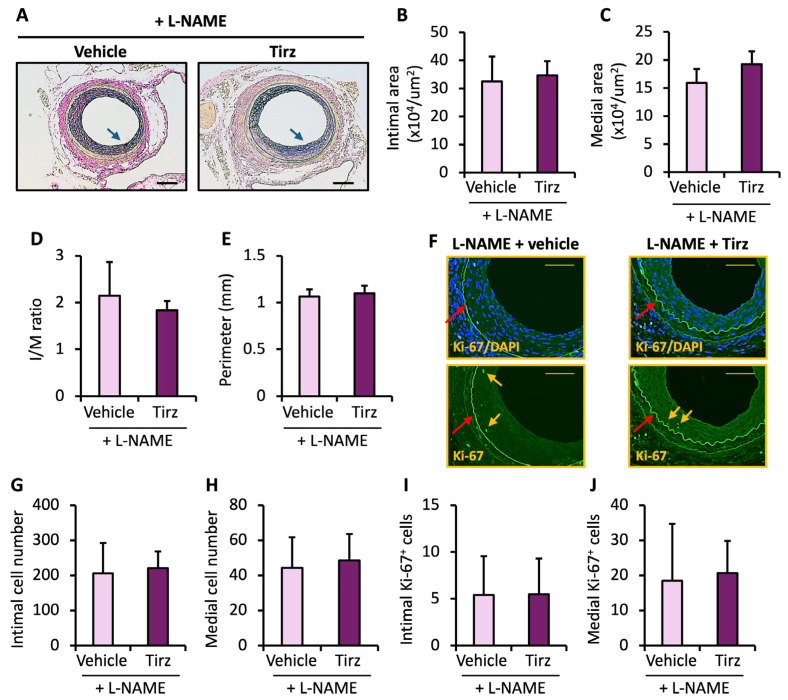
**Effects of tirzepatide on arterial remodeling in non-diabetic mice co-treated with L-NAME.** Injured arteries were harvested from non-diabetic mice treated with L-NAME alone or L-NAME + tirzepatide (1.0 nmol/kg/day), 24–26 days after wire injury: (**A**) Representative images of EVG-stained femoral arteries. Arrows indicate the intima. Scale bar = 100 μm. (**B**) Intimal area. (**C**) Medial area. (**D**) I/M ratio. (**E**) Arterial perimeter. (**B**–**E**) L-NAME alone, *n* = 6; L-NAME + tirzepatide, *n* = 7. (**F**) Representative images of femoral arteries immunostained for Ki-67 (green). Nuclei were counterstained with DAPI (blue). Yellow arrows indicate Ki-67-positive cells, and red arrows indicate the internal elastic lamina. Scale bar = 100 μm. (**G**,**H**) Total cell number in the intima and media, respectively. (**I**,**J**) Number of Ki-67-positive cells in the intima and media, respectively. (**G**–**J**) L-NAME alone, *n* = 4; L-NAME + tirzepatide, *n* = 4. Data are expressed as means ± SD. L-NAME, Nω-nitro-L-arginine methyl ester; Tirz, tirzepatide.

Next, to assess the role of endothelial NO in the protective effects of tirzepatide against arterial remodeling, mice were co-treated with the NOS inhibitor L-NAME. As shown in Table 2, physiological and biochemical parameters were comparable between groups. Under these conditions, the protective effects of tirzepatide on intimal hyperplasia or cell proliferation were abolished (Figure 2B–J; Supplemental Figure S1C,D).

### 3.3. Tirzepatide, but Not Semaglutide, Suppressed Intimal Hyperplasia and Cell Proliferation in Non-Diabetic Mice

**Figure 3:**
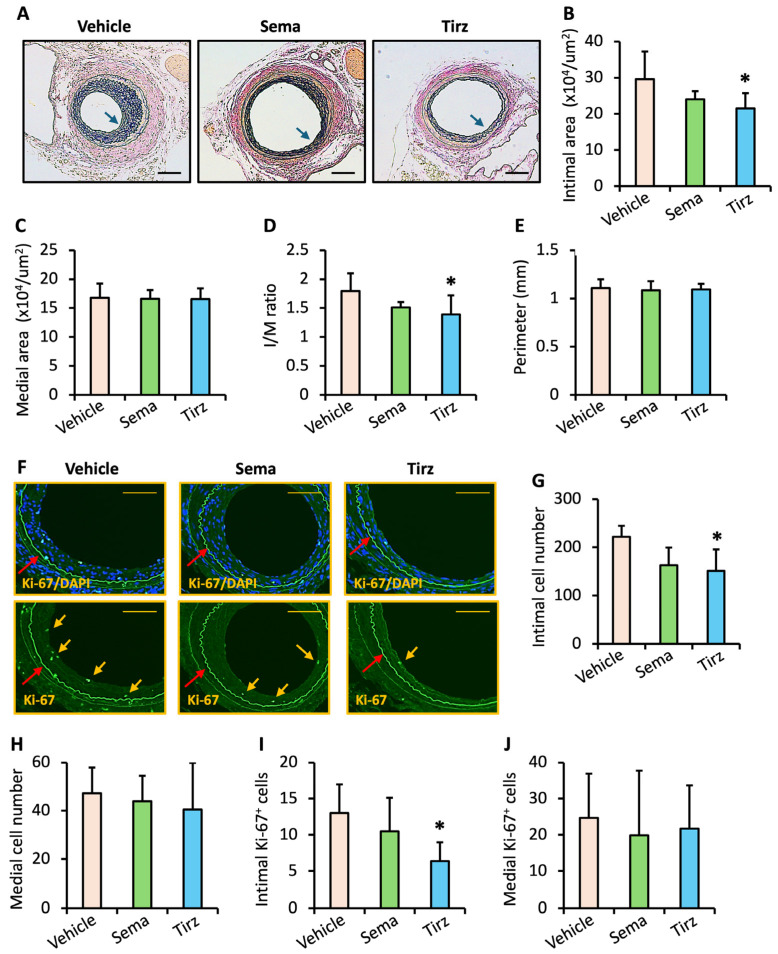
**Effects of semaglutide and tirzepatide on arterial remodeling in non-diabetic mice.** Injured arteries were harvested from non-diabetic mice treated with vehicle, semaglutide (1.0 nmol/kg/day), or tirzepatide (1 nmol/kg/day), 24–26 days after wire injury: (**A**) Representative images of EVG-stained femoral arteries. Arrows indicate the intima. Scale bar = 100 μm. (**B**) Intimal area. (**C**) Medial area. (**D**) I/M ratio. (**E**) Arterial perimeter. (**B**–**E**) vehicle, *n* = 8; semaglutide, *n* = 7; tirzepatide, *n* = 7. (**F**) Representative images of femoral arteries immunostained for Ki-67 (green). Nuclei were counterstained with DAPI (blue). Yellow arrows indicate Ki-67-positive cells, and red arrows indicate the internal elastic lamina. Scale bar = 100 μm. (**G**,**H**) Total cell number in the intima and media, respectively. (**I**,**J**) Number of Ki-67-positive cells in the intima and media, respectively. (**G**–**J**) Vehicle, *n* = 5; semaglutide, *n* = 5; tirzepatide, *n* = 5. Data are expressed as means ± SD. * *p* < 0.05 vs. vehicle group. Sema, semaglutide; Tirz, tirzepatide.

Third, the effects of tirzepatide on arterial remodeling were compared with those of semaglutide at an equivalent molar dose (1.0 nmol/kg/day) in non-diabetic mice. As presented in Table 3, tirzepatide significantly reduced fasting plasma glucose and triglyceride levels compared with the vehicle, whereas semaglutide significantly increased pulse rates. Morphological assessment revealed that tirzepatide significantly decreased intimal area, I/M ratio, and intimal total and proliferating cell numbers without altering other vascular parameters (Figure 3B–J; Supplemental Figure S1E,F), consistent with the findings of our first experiment. In contrast, semaglutide did not significantly affect any of the vascular parameters examined (Figure 3B–J; Supplemental Figure S1E,F).

### 3.4. Tirzepatide and Semaglutide Similarly Suppressed Intimal Hyperplasia and Cell Proliferation in Diabetic Mice

**Figure 4:**
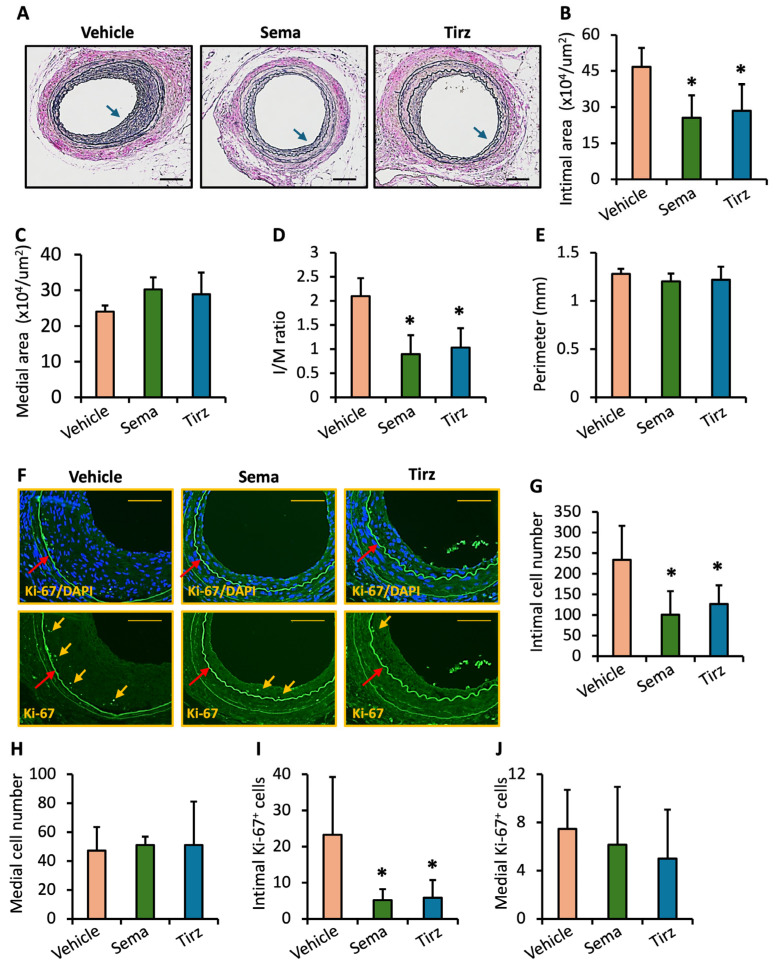
**Effects of semaglutide and tirzepatide on arterial remodeling in diabetic mice.** Injured arteries were harvested from diabetic mice treated with vehicle, semaglutide (1.0 nmol/kg/day), or tirzepatide (1.0 nmol/kg/day), 24–26 days after wire injury: (**A**) Representative images of EVG-stained femoral arteries. Arrows indicate the intima. Scale bar = 100 μm. (**B**) Intimal area. (**C**) Medial area. (**D**) I/M ratio. (**E**) Arterial perimeter. (**B**–**E**) Vehicle, *n* = 5; semaglutide, *n* = 6; tirzepatide, *n* = 6. (**F**) Representative images of femoral arteries immunostained for Ki-67 (green). Nuclei were counterstained with DAPI (blue). Yellow arrows indicate Ki-67-positive cells, and red arrows indicate the internal elastic lamina. Scale bar = 100 μm. (**G**,**H**) Total cell number in the intima and media, respectively. (**I**,**J**) Number of Ki-67-positive cells in the intima and media, respectively. (**G**–**J**) Vehicle, *n* = 5; semaglutide, *n* = 5; tirzepatide, *n* = 5. Data are expressed as means ± SD. * *p* < 0.05 vs. vehicle group. Sema, semaglutide; Tirz, tirzepatide.

Fourth, the vasoprotective effects of tirzepatide and semaglutide were evaluated at an equivalent molar dose (1.0 nmol/kg/day) in diabetic mice. Both tirzepatide and semaglutide significantly reduced fasting plasma glucose levels and improved insulin sensitivity, as assessed by HOMA-IR (Table 4). Furthermore, both treatments similarly decreased intimal area, I/M ratio, and intimal total and proliferating cell numbers (Figure 4B,D,G,I), whereas neither treatment affected medial area, arterial perimeter, medial total and proliferating cell numbers, or intimal and medial cell densities (Figure 4C,E,H,J; Supplemental Figure S1G,H). Consistent with the findings in non-diabetic mice, the numbers of total and proliferating cells in the intima were significantly correlated with intimal area (Supplemental Table S3).

### 3.5. Tirzepatide and Liraglutide Similarly Increased NO Production in HUVECs

**Figure 5:**
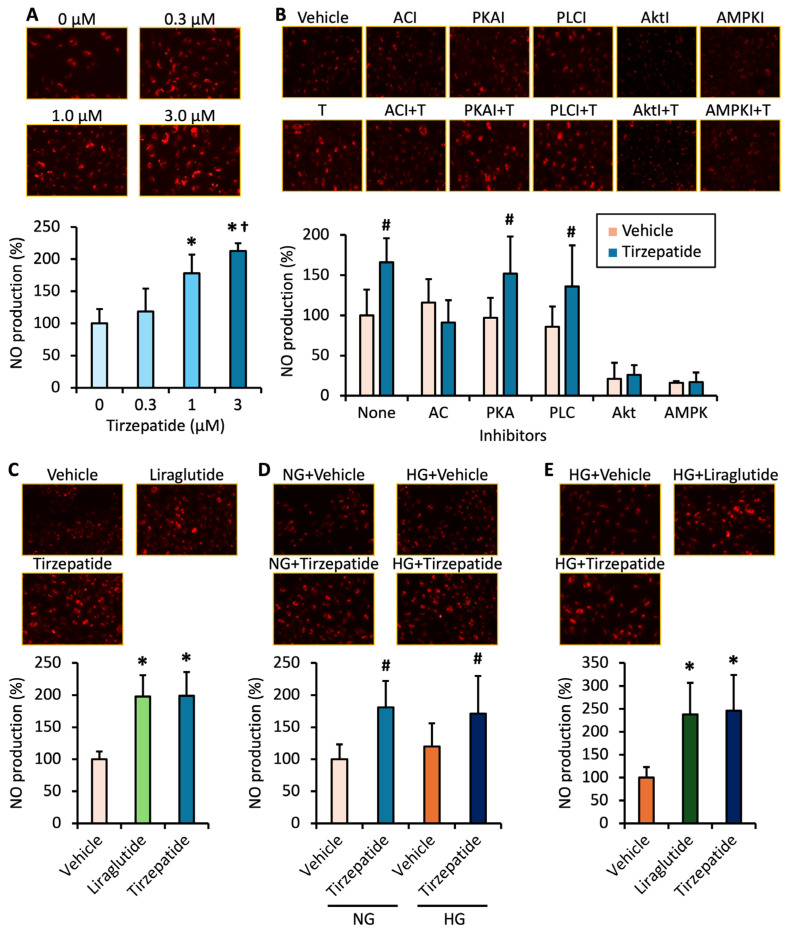
**Effect of tirzepatide on NO production in HUVECs under normal- and high-glucose conditions:** (**A**) Dose-dependent effects of tirzepatide on NO production. (**B**) Effects of pharmacological inhibitors on tirzepatide-stimulated NO production. (**C**) Comparison of the effects of tirzepatide and liraglutide on NO production. (**D**) Effects of tirzepatide on NO production under normal-glucose (5.5 mmol/L) and high-glucose (20 mmol/L) conditions. (**E**) Comparison of the effects of tirzepatide and liraglutide on NO production under high-glucose conditions. Representative fluorescence images of HUVECs obtained using the DAX-J2 probe are shown above each graph. Tirzepatide and liraglutide were used at 3 μmol/L for stimulation in (**B**–**E**). Data are expressed as means ± SD; *n* = 4–8 per group. *, *p* < 0.05 vs. 0 μmol/L or vehicle; †, *p* < 0.05 vs. 0.3 μmol/L; #, *p* < 0.05 vs. the corresponding vehicle-treated group. ACI, adenylate cyclase inhibitor; PKAI, protein kinase A inhibitor; PLCI, phospholipase C inhibitor; AktI, Akt inhibitor; AMPKI, AMP-activated protein kinase inhibitor; T, tirzepatide; NG, normal glucose; HG, high glucose.

Finally, the effects of tirzepatide on endothelial NO production were examined in vitro. Stimulation with tirzepatide increased NO production in HUVECs in a dose-dependent manner (Jonckheere–Terpstra trend test, *p* < 0.05; Figure 5A). The tirzepatide-induced increase in NO production was abolished by AC, Akt, and AMPK inhibition but was unaffected by PKA or PLC inhibition (Figure 5B). Western blot analyses further showed that tirzepatide increased p-eNOS levels and tended to increase the p-eNOS/t-eNOS ratio, whereas total eNOS expression was unchanged (Supplemental Figure S2A–D). Tirzepatide also increased p-AMPK levels and the p-AMPK/t-AMPK ratio, with these changes occurring earlier than the increase in p-eNOS (Supplemental Figure S2E–H). In contrast, p-Akt was not reproducibly detected under the present conditions. At equivalent molar concentrations, tirzepatide and liraglutide similarly increased NO production (Figure 5C). Furthermore, tirzepatide-induced NO production was preserved under hyperglycemic conditions (Figure 5D) and was comparable with that of liraglutide (Figure 5E).

## 4. Discussion

Our previous studies demonstrated that activation of GLP-1R or GIPR attenuates arterial remodeling and atherosclerosis independently of glucose lowering in experimental models of cardiovascular disease [5,6,7,8,9]. However, whether dual GIPR/GLP-1R activation confers similar vascular protection and how its effects compare with those of GLP-1R agonists alone have remained unclear. In the present study, we demonstrated for the first time that tirzepatide, a dual GIPR/GLP-1R agonist, suppresses wire injury-induced arterial remodeling through an NO-dependent mechanism, at least partly independent of glucose reduction. Furthermore, tirzepatide, but not semaglutide, exerted vasoprotective effects in non-diabetic mice, whereas both agents showed comparable protective effects in diabetic mice.

In the dose–response experiment of non-diabetic mice, we found that the low dose (0.16 nmol/kg/day) suppressed arterial remodeling without affecting plasma glucose levels or other metabolic parameters. Although direct comparison between rodents and humans should be interpreted with caution because of species differences in pharmacokinetics [29], our findings support the possibility that tirzepatide may confer vasoprotection at doses lower than those required for glucose-lowering actions.

Tirzepatide and semaglutide have been shown to reduce body weight in both animals and humans, regardless of diabetes status [15,16,21,22]. However, in the present study, even higher doses of tirzepatide (1.0 and 6.0 nmol/kg/day) did not affect body weight gain despite significantly lowering plasma glucose levels. We previously reported that body weight gain is impaired for several weeks after wire injury, possibly because of surgical stress and chronic vascular inflammation [6], which may have masked the weight-lowering effects of tirzepatide. Therefore, the protective effects of tirzepatide against arterial remodeling observed in the present study are unlikely to be attributable to changes in body weight.

Endothelial NO suppresses excessive cellular proliferation and thereby limits arterial remodeling [30]. In the present study, tirzepatide increased NO production from HUVECs at concentrations of 0.3–3.0 μmol/L. Because peak plasma concentrations after the highest approved clinical dose of tirzepatide (15 mg/week) have been reported to reach approximately 3000 ng/mL (0.6 μmol/L) [31], this concentration range includes clinically relevant levels. In vivo, the NOS inhibitor L-NAME abolished the inhibitory effects of tirzepatide on arterial remodeling and intimal cell proliferation, consistent with our previous findings for liraglutide and GIP [6,7]. In addition, intimal hyperplasia was closely associated with cell proliferation, rather than cell density. These findings suggest that tirzepatide limits neointimal formation mainly by enhancing endothelial NO signaling and suppressing intimal cell proliferation.

GIPR and GLP-1R primarily signal through Gs-mediated AC activation and cAMP production, although PLC signaling and intracellular Ca^2+^ mobilization can also be activated in some cell types [32,33]. In HUVECs, inhibition of AC, but not PLC, abolished tirzepatide-induced NO release. AMPK inhibition also blocked this response. Consistently, Western blot analyses showed increased phosphorylation of AMPK and eNOS, supporting the involvement of an AC–AMPK–eNOS pathway [34,35]. Akt inhibition also reduced NO release, suggesting possible functional involvement of Akt-related signaling [36,37,38,39]. However, reproducible detection of Akt phosphorylation could not be achieved under our experimental conditions. Therefore, the precise role of Akt signaling requires further investigation.

Intimal hyperplasia is an important pathological process involved in diabetic macrovascular disease and post-intervention restenosis [40]. The present results should be interpreted as mechanistic evidence suggesting that NO-dependent vascular protection may be one possible mechanism related to the cardiovascular benefits of tirzepatide, especially when endothelial dysfunction and reduced NO bioavailability are involved. At the same time, the wire injury model does not fully mimic the complex pathogenesis of human cardiovascular diseases. In addition to endothelial NO signaling, other vascular cells may also be involved in GIP/GIPR-mediated vascular protection [9]. For example, GIP has been reported to suppress the proliferation of vascular smooth muscle cells. GIP/GIPR signaling in macrophages may also reduce foam cell formation and inflammatory activation, as reviewed previously [9]. These mechanisms may be relevant to vascular diseases in which vascular smooth muscle cell proliferation and inflammation contribute to disease progression. Further studies using additional disease models, such as atherosclerosis models, are required to establish the clinical relevance of these findings.

To determine whether dual GIPR/GLP-1R agonism provides vascular benefits beyond GLP-1R agonism alone, we compared tirzepatide with semaglutide in mice and with liraglutide in HUVECs. In vitro, tirzepatide and liraglutide at equivalent molar concentrations similarly increased NO production under both normal- and high-glucose conditions. However, tirzepatide, but not semaglutide, showed vasoprotective effects in non-diabetic mice. These findings suggest that the difference between tirzepatide and semaglutide in vivo cannot be explained only by their direct effects on endothelial NO production. Additional vascular actions related to GIPR activation may contribute to the effect of tirzepatide under non-diabetic conditions, although receptor-specific experiments were not performed in the present study.

In contrast, tirzepatide and semaglutide similarly suppressed arterial remodeling in diabetic mice. This comparable effect is unlikely to be explained by reduced GIPR expression, because our previous study showed that arterial GIPR expression was not decreased in diabetic mice [7]. A previous study has also shown that insulin resistance, rather than hyperglycemia itself, promotes arterial remodeling after wire injury in rodent models [41]. In the present study, both tirzepatide and semaglutide improved insulin resistance in diabetic mice. Therefore, the improvement of insulin resistance by both agents may have contributed to their similar protective effects under diabetic conditions. In addition, our previous study showed that wire injury-induced vascular inflammation is enhanced in diabetic mice and that liraglutide suppresses this inflammatory response in diabetic, but not non-diabetic, mice [6]. On the other hand, GIP shows no effect on vascular inflammation in either non-diabetic or diabetic mice [7]. Thus, diabetes-associated vascular inflammation may be particularly responsive to GLP-1R signaling. This may explain why semaglutide exerted a clear protective effect in diabetic mice, whereas its effect was limited in non-diabetic mice, in which the GLP-1R-sensitive inflammatory component may be less prominent. These metabolic and inflammatory effects may have reduced the apparent difference between tirzepatide and semaglutide in diabetic mice. However, because inflammatory markers were not directly measured in the injured arteries in the present study, this interpretation remains speculative.

There are several limitations to the present study. First, tirzepatide and semaglutide were continuously administered to mice because of differences in pharmacokinetics between humans and rodents [29]. Although the subcutaneous route of administration was maintained, this delivery method differs from the once-weekly regimen used in humans [11,12,13]. In addition, pharmacokinetic parameters were not directly measured; therefore, the relationship between drug exposure and vascular protection remains unclear, which may limit the translational interpretation of these findings. Second, although tirzepatide significantly suppressed arterial remodeling in non-diabetic and diabetic mice, we did not directly determine the relative contribution of GIPR and GLP-1R signaling to these effects. Furthermore, in vivo mechanistic validation was also limited because inflammatory markers and downstream signaling pathways were not directly assessed in injured arteries. Third, only male mice were used in the present study, and potential sex differences in the vascular effects of tirzepatide were not examined. Finally, we evaluated arterial remodeling at a single time point after vascular injury and did not assess long-term vascular outcomes. These limitations should be addressed in future studies to better define the translational relevance and mechanisms of tirzepatide-mediated vascular protection.

## 5. Conclusions

The present findings suggest that tirzepatide exerts vasoprotective effects, at least in part, through stimulation of endothelial NO production. At equivalent molar doses, tirzepatide, but not semaglutide, exerted vasoprotective effects in non-diabetic mice, whereas both agents exhibited comparable effects in diabetic mice.

## Figures and Tables

**Table 1:** Physiological and biochemical parameters of non-diabetic mice treated with different doses of tirzepatide.

	Vehicle	0.16 nmol/kg/day	1.0 nmol/kg/day	6.0 nmol/kg/day
Number of mice	14	8	7	7
Initial body weight (g)	26.2 ± 1.3	27.2 ± 1.3	26.4 ± 1.3	27.7 ± 1.8
Final body weight (g)	25.6 ± 1.3	26.1 ± 1.1	25.3 ± 1.4	26.6 ± 1.0
Body weight change (g)	−0.6 ± 0.9	−1.0 ± 0.6	−1.1 ± 0.6	−1.2 ± 1.6
Plasma glucose (mg/dL)	146 ± 13	150 ± 12	105 ± 19 ^ab^	103 ± 12 ^ab^
Plasma insulin (ng/mL)	0.16 ± 0.15	0.11 ± 0.10	0.16 ± 0.13	0.10 ± 0.13
HOMA-β	0.66 ± 0.63	0.47 ± 0.44	1.56 ± 1.56	1.02 ± 1.26
HOMA-IR	0.06 ± 0.06	0.04 ± 0.04	0.04 ± 0.03	0.03 ± 0.04
Plasma total cholesterol (mg/dL)	79 ± 11	71 ± 14	75 ± 9	76 ± 5
Plasma triglycerides (mg/dL)	105 ± 6	99 ± 6	98 ± 14	96 ± 5

Data are expressed as means ± SD. HOMA-β, homeostasis model assessment of β-cell function; HOMA-IR, homeostasis model assessment of insulin resistance. *p* < 0.05: ^a^, vs. vehicle group; ^b^, vs. 0.16 nmol/kg/day group.

**Table 2:** Physiological and biochemical parameters of non-diabetic mice treated with L-NAME alone or L-NAME plus tirzepatide.

	L-NAME	L-NAME + Tirzepatide
Number of mice	6	7
L-NAME intake (mg/kg/day)	36.0 ± 13.8	32.3 ± 8.7
Initial body weight (g)	26.2 ± 1.6	26.7 ± 0.9
Final body weight (g)	25.4 ± 0.9	25.7 ± 0.7
Body weight change (g)	−0.8 ± 0.9	−1.0 ± 0.7
Plasma glucose (mg/dL)	153 ± 15	138 ± 24
Plasma insulin (ng/mL)	0.33 ± 0.42	0.23 ± 0.31
HOMA-β	1.53 ± 2.00	1.41 ± 1.82
HOMA-IR	0.12 ± 0.14	0.07 ± 0.10
Plasma total cholesterol (mg/dL)	77 ± 15	82 ± 14
Plasma triglycerides (mg/dL)	103 ± 12	93 ± 3

Data are expressed as means ± SD. Tirzepatide was administered at 1.0 nmol/kg/day. L-NAME, Nω-nitro-L-arginine methyl ester.

**Table 3:** Physiological and biochemical parameters of non-diabetic mice treated with vehicle, semaglutide, or tirzepatide.

	Vehicle	Semaglutide	Tirzepatide
Number of mice	8	7	7
Initial body weight (g)	26.8 ± 1.5	27.1 ± 1.8	26.9 ± 1.8
Final body weight (g)	27.8 ± 1.0	28.9 ± 1.4	28.5 ± 1.5
Body weight change (g)	1.1 ± 0.8	1.8 ± 0.7	1.6 ± 0.8
Pulse rates (beats/min)	644 ± 98	747 ± 20 ^a^	708 ± 72
Systolic blood pressure (mmHg)	127 ± 15	122 ± 15	123 ± 13
Plasma glucose (mg/dL)	153 ± 28	147 ± 22	116 ± 20 ^a^
Plasma insulin (ng/mL)	0.17 ± 0.16	0.16 ± 0.19	0.09 ± 0.14
HOMA-β	0.65 ± 0.53	0.82 ± 1.06	1.02 ± 2.01
HOMA-IR	0.07 ± 0.07	0.06 ± 0.07	0.02 ± 0.03
Plasma total cholesterol (mg/dL)	72 ± 13	78 ± 8	80 ± 8
Plasma triglycerides (mg/dL)	107 ± 11	105 ± 9	93 ± 6 ^a^

Data are expressed as means ± SD. *p* < 0.05: ^a^, vs. vehicle group. Semaglutide and tirzepatide were administered at 1.0 nmol/kg/day.

**Table 4:** Physiological and biochemical parameters of diabetic mice treated with vehicle, semaglutide, or tirzepatide.

	Vehicle	Semaglutide	Tirzepatide
Number of mice	5	6	6
Initial body weight (g)	45.2 ± 2.0	44.9 ± 1.7	47.0 ± 2.3
Final body weight (g)	46.9 ± 1.8	47.4 ± 1.8	48.3 ± 1.8
Body weight change (g)	1.7 ± 0.9	2.5 ± 1.0	1.3 ± 0.8
Pulse rates (beats/min)	699 ± 107	664 ± 67	678 ± 99
Systolic blood pressure (mmHg)	117 ± 15	113 ± 24	102 ± 15
Plasma glucose (mg/dL)	161 ± 31	109 ± 25 ^a^	106 ± 16 ^a^
Plasma insulin (ng/mL)	1.9 ± 0.4	1.1 ± 0.3	1.0 ± 0.8
HOMA-β	7.1 ± 1.8	8.8 ± 5.9	11.2 ± 11.3
HOMA-IR	0.76 ± 0.27	0.34 ± 0.17 ^a^	0.26 ± 0.18 ^a^
Plasma total cholesterol (mg/dL)	137 ± 5	122 ± 15	134 ± 12
Plasma triglycerides (mg/dL)	192 ± 32	176 ± 46	168 ± 12

Data are expressed as means ± SD. *p* < 0.05: ^a^, vs. vehicle group. Semaglutide and tirzepatide were administered at 1.0 nmol/kg/day.

## Data Availability

The raw data supporting the conclusions of this article will be made available by the authors upon request.

## Supplementary Materials

The following supporting information can be downloaded at: https://www.mdpi.com/article/10.3390/biomedicines14071554/s1.

## Abbreviations

The following abbreviations are used in this manuscript:

AC	Adenylate cyclase
AMPK	AMP-activated protein kinase
ASCVD	Atherosclerotic cardiovascular disease
bFGF	Basic fibroblast growth factor
DAPI	4′,6-Diamidino-2-phenylindole
eNOS	Endothelial nitric oxide synthase
EVG	Elastica van Gieson
FBS	Fetal bovine serum
GIP	Glucose-dependent insulinotropic polypeptide
GIPR	Glucose-dependent insulinotropic polypeptide receptor
GLP-1	Glucagon-like peptide-1
GLP-1R	Glucagon-like peptide-1 receptor
GLP-1RA	Glucagon-like peptide-1 receptor agonist
HOMA-β	Homeostasis model assessment of β-cell function
HOMA-IR	Homeostasis model assessment of insulin resistance
HUVEC	Human umbilical vein endothelial cell
IEL	Internal elastic lamina
I/M ratio	Intima-to-media ratio
L-NAME	Nω-nitro-L-arginine methyl ester
NO	Nitric oxide
NOS	Nitric oxide synthase
PBS	Phosphate-buffered saline
PKA	Protein kinase A
PLC	Phospholipase C
SD	Standard deviation
VEC	Vascular endothelial cell
