# Supplementary material for: Tirzepatide Attenuates Wire Injury-Induced Arterial Remodeling in Non-Diabetic and Diabetic Mice: Comparison with Semaglutide
Source: Biomedicines. 2026 Jul 11;14(7):1554. doi: 10.3390/biomedicines14071554 (PMC13405626; doi:10.3390/biomedicines14071554)
Supplement: Supplementary file 1 [file biomedicines-14-01554-s001.zip › biomedicines-4403923-supplementary.pdf]

Supplemental Table S1. Correlation among vascular parameters in non-diabetic mice.

	Intimal area	Medial area	Intimal cell number	Medial cell number	Intimal Ki67$^+$ cells	Medial Ki67$^+$ cells
Intimal area	1.00					
Medial area	0.49*	1.00				
Intimal cell number	0.83***	0.34	1.00			
Medial cell number	0.26	0.52*	0.44*	1.00		
Intimal Ki67$^+$ cells	0.58**	0.02	0.68***	0.10	1.00	
Medial Ki67$^+$ cells	0.43	−0.02	0.33	−0.57	0.50*	1.00

r values indicate Pearson's correlation coefficients. *, $p < 0.05$; **, $p < 0.01$; ***, $p < 0.001$. n = 21 (9 and 12 sections from vehicle and tirzepatide 1.0 nmol/kg/day groups, respectively).

Supplementary Table S2. Mean differences and 95% confidence intervals for key vascular outcomes.

Experiment	Outcome	Comparison	Mean difference (95% CI)	p value
1	Intimal area	Vehicle vs Tirz 0.16	8.29 (0.26 to 16.31)	0.041
1	Intimal area	Vehicle vs Tirz 1.0	9.66 (0.85 to 18.47)	0.028
1	Intimal area	Vehicle vs Tirz 6.0	13.70 (5.33 to 22.07)	<0.001
1	Intimal area	Tirz 0.16 vs Tirz 6.0	5.41 (-3.83 to 14.65)	0.398
1	Intimal area	Tirz 1.0 vs Tirz 6.0	4.04 (-5.89 to 13.97)	0.689
1	Intimal area	Tirz 0.16 vs Tirz 1.0	1.37 (-8.27 to 11.01)	0.980
1	I/M ratio	Vehicle vs Tirz 0.16	0.54 (0.04 to 1.03)	0.031
1	I/M ratio	Vehicle vs Tirz 1.0	0.73 (0.18 to 1.28)	0.005
1	I/M ratio	Vehicle vs Tirz 6.0	0.80 (0.29 to 1.32)	0.001
1	I/M ratio	Tirz 0.16 vs Tirz 6.0	0.27 (-0.31 to 0.84)	0.588
1	I/M ratio	Tirz 0.16 vs Tirz 1.0	0.19 (-0.40 to 0.79)	0.814
1	I/M ratio	Tirz 1.0 vs Tirz 6.0	0.07 (-0.54 to 0.69)	0.988
2	Intimal area	Vehicle vs Tirz	-0.19 (-9.13 to 8.76)	0.965
2	I/M ratio	Vehicle vs Tirz	0.38 (-0.20 to 0.97)	0.180
3	Intimal area	Vehicle vs Tirz	8.05 (1.02 to 15.09)	0.023
3	Intimal area	Vehicle vs Sema	5.54 (-1.50 to 12.57)	0.140
3	Intimal area	Sema vs Tirz	2.52 (-4.75 to 9.79)	0.659
3	I/M ratio	Vehicle vs Tirz	0.40 (0.05 to 0.75)	0.024
3	I/M ratio	Vehicle vs Sema	0.28 (-0.07 to 0.63)	0.138
3	I/M ratio	Sema vs Tirz	0.12 (-0.24 to 0.49)	0.672
4	Intimal area	Vehicle vs Sema	21.16 (5.93 to 36.39)	0.007
4	Intimal area	Vehicle vs Tirz	18.28 (3.04 to 33.51)	0.019
4	Intimal area	Tirz vs Sema	2.88 (-11.64 to 17.41)	0.863
4	I/M ratio	Vehicle vs Sema	1.20 (0.58 to 1.82)	<0.001
4	I/M ratio	Vehicle vs Tirz	1.07 (0.45 to 1.69)	0.001
4	I/M ratio	Tirz vs Sema	0.13 (-0.46 to 0.72)	0.826

Data are presented as mean difference (95% confidence interval). Mean differences were calculated as the first group minus the second group in each comparison, and positive values indicate higher values in the first group. Tirz, tirzepatide; Sema, semaglutide; I/M ratio, intima/media ratio.

Supplemental Table S3. Correlation among vascular parameters in diabetic mice.

	Intimal area	Medial area	Intimal cell number	Medial cell number	Intimal $Ki67^+$ cells	Medial $Ki67^+$ cells
Intimal area	1.00					
Medial area	−0.44*	1.00				
Intimal cell number	0.96***	−0.43*	1.00			
Medial cell number	0.07	0.64***	0.09	1.00		
Intimal $Ki67^+$ cells	0.63***	−0.54**	0.67***	−0.21	1.00	
Medial $Ki67^+$ cells	0.16	−0.35	0.13	−0.34	0.47	1.00

r values indicate Pearson's correlation coefficients. *, $p < 0.05$; **, $p < 0.01$; ***, $p < 0.001$. n = 30 (10 sections each from vehicle, semaglutide, and tirzepatide groups).

Supplemental Figure S1. Intimal and medial cell densities in non-diabetic and diabetic mice treated with vehicle, tirzepatide, or semaglutide.

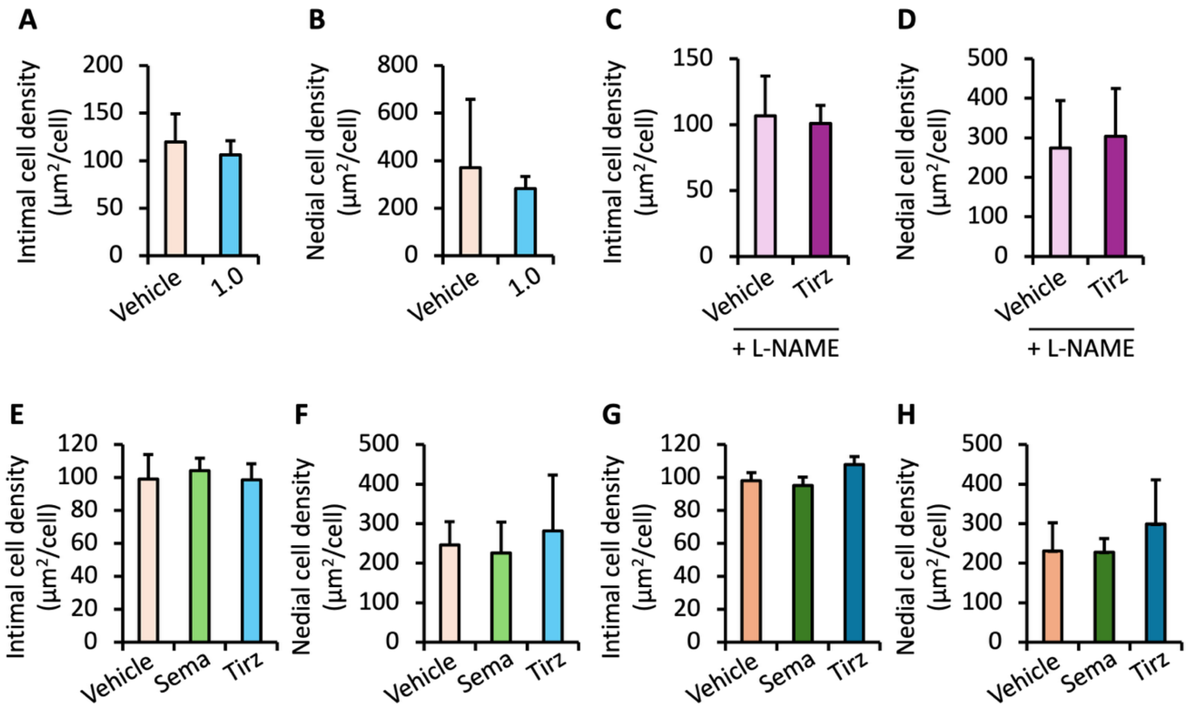

(A, B) Effects of tirzepatide on intimal and medial cell densities in non-diabetic mice (vehicle, *n* = 4; tirzepatide 1.0 nmol/kg/day, *n* = 4). (C, D) Effects of tirzepatide on intimal and medial cell densities in non-diabetic mice treated with L-NAME (L-NAME alone, *n* = 4; L-NAME + tirzepatide, *n* = 4). (E, F) Comparison of the effects of tirzepatide and semaglutide on intimal and medial cell densities in non-diabetic mice (vehicle, *n* = 5; semaglutide, *n* = 5; tirzepatide, *n* = 5). (G, H) Comparison of the effects of tirzepatide and semaglutide on intimal and medial cell densities in diabetic mice (vehicle, *n* = 5; semaglutide, *n* = 5; tirzepatide, *n* = 5). Cell density was calculated as the ratio of area to cell number and is expressed as μm²/cell. Data are expressed as mean ± SD.

Supplemental Figure S2. Protein levels of phosphorylated and total eNOS and AMPK after tirzepatide stimulation in HUVECs.

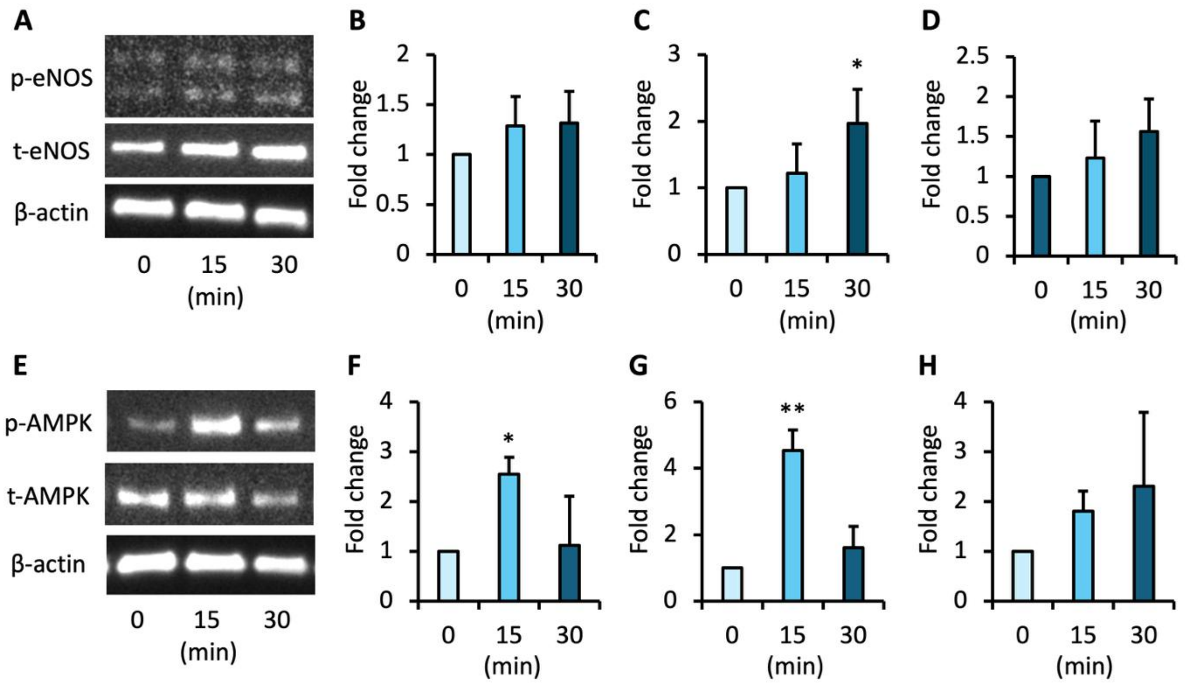

(A–D) Effects of tirzepatide on phosphorylated and total eNOS levels. (A) Representative immunoblot bands. (B) p-eNOS levels normalized to total eNOS levels. (C) p-eNOS levels normalized to β-actin levels. (D) total eNOS levels normalized to β-actin levels. (E–H) Effects of tirzepatide on phosphorylated and total AMPK levels. (E) Representative immunoblot bands. (F) p-AMPK levels normalized to total AMPK levels. (G) p-AMPK levels normalized to β-actin levels. (H) total AMPK levels normalized to β-actin levels. Data are expressed as fold changes relative to 0 min. $n = 3$ per group. Data are expressed as mean ± SD. One-way ANOVA followed by Dunnett's test. *, $p < 0.05$; **, $p < 0.01$ vs. 0 min.
